# Surgical Outcomes and Predictive Factor Analysis for Facial Nerve Preservation in Patients With Cerebellopontine Angle (CPA) Tumors: A Ten-Year Single Institutional Study

**DOI:** 10.7759/cureus.61756

**Published:** 2024-06-05

**Authors:** Ravi P Verma, Awdhesh Yadav, Vijendra Kumar, B K Ojha, Anil Chandra, Rajat Verma

**Affiliations:** 1 Neurosurgery, King George's Medical University, Lucknow, IND; 2 Neurosurgery, Kalyan Singh Super Speciality Cancer Institute and Hospital, Lucknow, IND

**Keywords:** vestibular schwannoma, retromastoid, meningioma, subtotal resection, cerebellopontine angle tumour

## Abstract

Objective

To analyze the surgical outcome and predictive factors for facial nerve preservation in patients with surgically operated cerebellopontine angle (CPA) tumors.

Methodology

Methodology Data were retrospectively retrieved from inpatient medical records of patients admitted with CPA tumors from January 1, 2011, to December 31, 2020, at our institute. Epidemiological, clinical and radiological findings, histopathological types, surgical outcomes, and facial nerve function of these patients were recorded using a data-gathering tool.

Results

Out of 230 patients, 188 (81.7%) were diagnosed histopathologically with vestibular schwannoma (VS), 20 (8.7%) with meningioma, 15 (6.5%) with epidermoid, and 7 (3.1%) with other conditions. The most common clinical features were hearing loss in VS and headaches in meningioma and epidermoid. Preoperatively, 103 (44.8%) had grade 2, 68 (29.6%) had grades 3 or 4, and 8 (3.5%) had grade 5 facial nerve palsy, while post-operatively, 93 (40.9%) patients had grade 2, 83 (36.6%) had grades 3 or 4, and 6 (2.6%) had grade 5 facial palsy. Greater facial nerve preservation was observed in patients with tumor sizes <4 cm (p=0.0041) and in those who underwent near-total (NTR) or subtotal resection (STR) (p=0.0442). Excellent facial nerve outcomes (HB grades 1 or 2) were noted in patients who underwent intraoperative facial nerve monitoring (p<0.0001). CSF leak and meningitis were present in 3.5% and 2.2% of patients, respectively. The mortality rate was 6.1%, with a recurrence rate of 4.8%.

Conclusion

Intraoperative facial nerve monitoring, tumor size less than 4 cm, and extent of resection (NTR/STR) are predictive factors that significantly affect facial nerve outcomes.

## Introduction

Of all the intracranial tumors, 10-15% are cerebellopontine angle (CPA) tumors, which are most commonly located in the posterior fossa of the cranial cavity [1-3]. They are usually benign, with the majority (80-94%) being vestibular schwannomas (VS). The other types of CPA tumors include meningiomas (3-10%), epidermoids (2-4%), other cranial nerve schwannomas, cholesteatomas, and metastases (less than 2%) [1-3].

The most frequently witnessed symptoms of CPA tumors include hearing loss, headache, dizziness, ataxia, tinnitus, vertigo, and dysfunction of the cranial nerves [4]. Surgery is considered the optimal treatment for CPA tumors after proper preoperative planning, except for small tumors (<2.5 cm), which can be managed by radiosurgery [5].

Surgical outcomes and factors predicting facial nerve morbidity and severity have rarely been specifically studied in North India, especially in our region. Preoperative facial nerve status, tumor size, and extent of resection majorly affect facial nerve morbidity in the immediate postoperative period. To the best of our knowledge, only a few studies [6-8] in North India have correlated these factors with facial nerve outcomes.

Our center, being a tertiary care center, caters to a large population of Uttar Pradesh, including parts of Uttarakhand, Bihar, and Nepal. Therefore, this study aims to analyze the epidemiological and clinico-radiological characteristics, extent of resection, surgical outcomes, and predictive factors for facial nerve preservation in CPA tumors.

## Materials and methods

A retrospective observational study was conducted in the Department of Neurosurgery at King George's Medical University, Lucknow, between January 1, 2011, and December 31, 2020. The King George's Medical University, Uttar Pradesh (UP), Institutional Ethics Committee reviewed the research article in its meeting held on April 2, 2022, and granted approval with number VII-PGTSC-IIA/P4. The study examined the inpatient medical records of 345 patients. Cases with incomplete records and missing data were excluded. Two hundred thirty patients underwent final surgical intervention for their CPA tumors, and 115 patients were lost after CSF diversion. Ultimately, 230 patients were included in the study. The collected data were analyzed based on epidemiological, clinical, radiological, surgical outcomes, histopathological types, facial nerve dysfunction, and perioperative complications. Patients recruited for the study were followed up, and their clinical data, including facial nerve function, were recorded. Nineteen of these patients underwent intraoperative facial nerve monitoring. Thus, patients were also divided based on whether they were monitored intraoperatively or did not undergo intraoperative monitoring. Data were also evaluated for these two groups. The clinical information of the study subjects, including facial nerve function assessed by House and Brackmann grading (HB grading), was evaluated. These individuals underwent brain contrast-enhanced computed tomography (CECT) and MRI with contrast enhancement (CEMRI) in follow-up. The Chi-square test was used to analyze categorical variables, and a student t-test was used to compute a continuous variable. To assess statistical significance at the 5% level of significance, probability (p) was determined.

## Results

**Figure 1:**
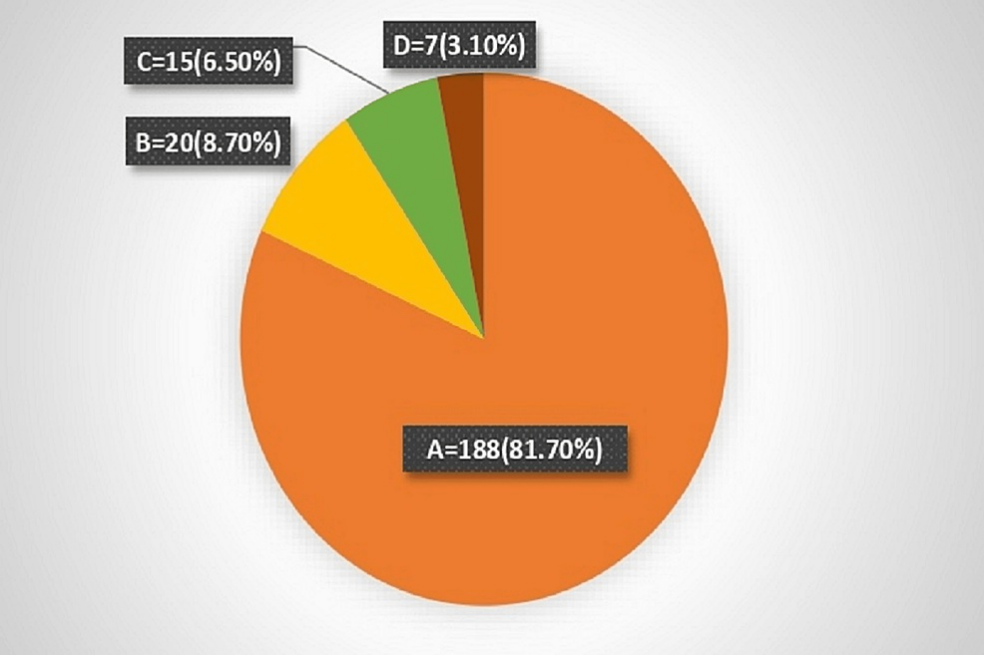
Distribution of tumors according to the histological types. A: Vestibular schwannoma B: Meningioma C: Epidermoid D: Others

Out of 230 patients, the predominant histopathology was VS, accounting for 188 (81.7%) patients, followed by 20 (8.7%) with meningioma, 15 (6.5%) with epidermoid, and 7 (3.1%) with other conditions (Figure 1). A majority, 94.8%, were sporadic cases, with only 5.2% being NF-2 associated.

The mean age of the patients was 36.3 ± 14.2 years. Most patients with VS and epidermoid were males, while females predominated in meningiomas (Table 1).

**Table 1:** Baseline characteristics of patients based on histopathological types of CPA tumors. CPA: Cerebellopontine angle; ICP: Intracranial pressure.

Variables		Vestibular schwannoma (N=188) [N(%)]	Meningioma (N=20) (N(%))	Epidermoid (N=15) (N(%))
Age	0-10 years	0 (0%)	0 (0%)	0 (0%)
	11-20 years	13 (6.9%)	3 (15%)	3 (20%)
	21-30 years	61 (32.4%)	4 (20%)	5 (33.3%)
	31-40 years	44 (23.5%)	7 (35%)	6 (40%)
	41-50 years	43 (22.8%)	2 (10%)	1 (6.7)
	>50 years	27 (14.4%)	4 (20%)	0 (0%)
Gender	Male	97 (51.6%)	8 (40%)	8 (53.3%)
	Female	91 (48.4%)	12 (60%)	7 (46.7%)
Clinical Features*	Hearing Loss	188 (100%)	12 (60%)	6 (40%)
	Headache	171 (90.90%)	18 (90%)	8 (53.30%)
	Ataxia	154 (81.90%)	13 (65%)	4 (26.70%)
	Facial Nerve Involvement	165 (87.80%)	8 (40%)	4 (26.70%)
	Trigeminal Nerve Involvement	90 (47.90%)	6 (30%)	5 (33.30%)
	Lower Cranial Nerve Involvement	65 (34.60%)	4 (20%)	3 (20%)
	Raised ICP	152 (80.90%)	9 (45%)	0 (0%)
	Tinnitus	47 (25%)	2 (10%)	3 (20%)
	Vertigo	33 (17.60%)	0 (0%)	2 (13.30%)
*Multiple answers				

VS patients were more common in the second to third decades of life, followed by the third to fourth decades. Meningiomas and epidermoids were more commonly found in the third to fourth decades of life (Table 1).

The most common symptoms across all histological types were hearing loss and headache. Almost all patients with VS presented with hearing loss, compared to 12 (60%) patients with meningioma and 6 (40%) with epidermoid. Headache was reported in 171 (90.9%) patients with VS, 18 (90%) with meningioma, and 8 (53.3%) with epidermoid. Facial nerve involvement was noted in 165 (87.8%) patients with VS, 8 (40%) with meningioma, and 4 (26.7%) with epidermoid. Trigeminal nerve dysfunction was present in 90 (47.9%) patients with VS, 6 (30%) with meningioma, and 5 (33.3%) with epidermoid. Thirty-three (17.6%) patients with VS experienced vertigo, compared to 2 (13.3%) with epidermoid. Tinnitus was present in 47 (25%) patients with VS, 2 (10%) with meningioma, and 3 (20%) with epidermoid (Table 1).

One hundred fourteen patients were followed up with a median follow-up period of 6 years. Facial nerve function improved in 32 patients (28.1%), remained the same in 77 (67.6%), and deteriorated in 5 (4.4%) during the follow-up.

Radiological findings on CEMRI

VS were primarily (93.08%) hypointense on T1-weighted MRI, with none being isointense. On T2-weighted imaging, they were mostly hyperintense (80.85%), followed by heterogeneous hyperintensity in 11.17% and solid cystic lesions in 7.98%. Meningiomas were generally hypointense on T1-weighted (65%) but were the only tumor type among the three that was isointense on T1-weighted (20%). No meningiomas were solid cystic on T2-weighted imaging.

Epidermoids were 100% hypointense on T1-weighted and hyperintense on T2-weighted. Post-contrast, all patients with VS showed heterogeneous contrast enhancement, all meningiomas were broad-based and had homogenous contrast enhancement, while epidermoids did not enhance and showed restriction on diffusion-weighted imaging (DWI) images (Table 2). One hundred fifty-four (66.9%) patients with VS had internal acoustic meatus (IAM) widening, while all patients with meningiomas had broad-based tumors with dural enhancement.

**Table 2:** Radiological MRI imaging of histological types of CPA tumors. CPA: Cerebellopontine angle.

Variables		Vestibular schwannoma N (%)	Meningioma N (%)	Epidermoid N (%)
T1-weighted MRI	Hypointense	175 (93.08%)	13 (65%)	15 (100%)
	Hypo-isointense	13 (6.90%)	3 (15%)	0 (0%)
	Isointense	0 (0%)	4 (20%)	0 (0%)
T2-weighted MRI	Hyperintense	152 (80.85%)	18 (90%)	15 (100%)
	Heterogeneous hyperintense	21 (11.17%)	2 (10%)	0 (0%)
	Solid cystic	15 (7.98%)	0 (0%)	0 (0%)
Contrast MRI	Heterogeneous contrast enhancement	188 (100%)	0 (0%)	0 (0%
	Homogenous contrast enhancement	0 (0%)	20 (100%)	0 (0%)
	Non-contrast enhancement	0 (0%)	0 (0%)	15 (100%)

Complications and outcomes

Postoperatively, wound infection was observed in 11 (4.8%) patients. Of these, a CSF leak was present in 8 (3.5%) patients with tumor size > 4 cm. Five patients were managed conservatively with compression bandages and medical therapy (Acetazolamide), while three required lumbar drain placement. Meningitis developed in 5 (2.2%) patients, of which 3 died from it. Postoperative hematoma developed in 5 (2.2%) patients, four of whom required re-exploration. Twenty-six (11.5%) patients developed post-operative lower cranial nerve palsy; of these, 10 were tracheostomized, and in 3 patients, the palsy improved (Table 3).

**Table 3:** Distribution of study participants based on the type of resection, tumor size, and facial nerve preservation. *Three patients expired on post-operative day 0. **Extent of resection is defined based on post-operative CECT/CEMRI brain. **Gross total resection (GTR) is defined as no residual tumor on imaging. **Near total resection (NTR) is defined as a tumor remnant less than 2.5 cm². **Subtotal resection (STR) is defined as any tumor larger than that defined for NTR. **A p-value < 0.05 is considered significant.

Variables		Facial nerve preservation N (%)	No facial nerve preservation N (%)	P-value(<0.05 significant)
Type of resection (N=227)*	GTR** (N=163)	126 (77.3%)	37 (22.7%)	0.0442 (Significant)
	NTR** AND STR** (N=64)	57 (89.06%)	7 (10.93%)	
Tumor size (N=227)*	1-2.5 cm	5 (100%)	0 (0%)	0.0041 (Significant)
	2.6-4 cm (large)	103 (88.03%)	14 (11.96%)	
	>4 cm (giant)	75 (71.42%)	30 (28.57%)	

Fourteen patients died during this study (a mortality rate of 6.1%). Of these, 10 died during admission and 4 during follow-up. Three patients died in the immediate postoperative period due to cardiorespiratory arrest, three from meningitis, three from respiratory failure, and one from hydrocephalus. Four patients died during follow-up due to comorbidities. Eleven patients (4.8%) experienced a recurrence in our study; four were reoperated, three were sent for gamma knife surgery, and four were followed up with no clinical deterioration (Table 3).

Tumor size, resection, and facial nerve preservation

The mean tumor size of CPA tumors in this study was 2.7 cm, with a range of 1.2-7.5 cm. It was observed that the majority of tumors were either large (2.6-4 cm) or giant (>4 cm), accounting for 118 (51.3%) and 107 (46.5%) patients, respectively. Facial nerve preservation was greater in patients with tumors smaller than 4 cm, which was statistically significant (p-value = 0.0041) (Table 4).

**Table 4:** Complications and outcomes in CPA tumors. CPA: Cerebellopontine angle.

Variables		No. of patients	Percentage
Complications	Wound infection	11	4.8%
	CSF leak in drain	8	3.5%
	Meningitis	5	2.2%
	Postoperative hematoma	5	2.2%
	Post-operative lower cranial involvement	26	11.5%
Mortality		14	6.1%
Recurrence (N=11)	Re-operated	4	36.4%
	Gamma Knife	3	27.2%
	In Follow-up	4	36.4%

All patients with CPA tumors underwent surgery via the retromastoid suboccipital approach, except for one patient with VS, who was operated through the translabyrinthine approach. Two hundred fifteen (93.5%) patients underwent gross total resection (GTR) and near-total resection (NTR), while 15 (6.5%) patients underwent subtotal resection (STR). No statistically significant correlation between the type of resection and tumor size was noticed (p-value = 0.251). However, a statistically significant association of facial nerve preservation with the type of resection was observed (p-value = 0.0442) (Table 4).

Facial nerve status, preservation, and outcome in monitored and non-monitored patients

Preoperatively, 103 (44.8%) patients had grade 2, 68 (29.6%) had grade 3 or 4, and 8 (3.5%) had grade 5 facial nerve palsy. Postoperatively, 93 (40.9%) patients had grade 2, 83 (36.6%) had grade 3 and 4, and 6 (2.6%) had grade 5 facial palsy.

It was observed that preoperatively, the majority of patients who underwent facial nerve monitoring had excellent facial nerve status (HB grade 1 or 2), with 16 out of 19 patients. Postoperatively, the facial nerve grade deteriorated in only one patient from an excellent grade (HB grade 1 or 2) to an intermediate grade (HB grade 3 or 4).

Nineteen patients with CPA tumors underwent facial nerve monitoring, of which 18 (94.7%) achieved facial nerve preservation in the postoperative period, compared to 165 (79.3%) patients who were not monitored (p-value = 0.104). It was observed during the study that excellent facial nerve outcomes (HB grade 1 or 2) were higher in patients who underwent intraoperative facial nerve monitoring compared to those who did not (78.9% vs 59.1%), which was statistically significant (p-value < 0.0001). Intermediate (HB grade 3 or 4) and poor (HB grade 5 or 6) facial nerve outcomes were higher in non-monitored patients compared to monitored patients (37.9% and 21.9% versus 2.9% and 0%) (Table 5).

**Table 5:** Facial nerve status pre- and post-operative: preservation and outcome in monitored and non-monitored patients.

Variable		Monitored (N=19) N (%)	Non-monitored (Preop N=211; Postop N=208) N (%)	P-value (<0.05 significant)
Pre-operative facial nerve status (N=230)	Grade 1	4 (21.05%)	47 (22.27%)	0.296 (Not significant)
	Grade 2	12 (63.15%)	91 (43.12%)	
	Grade 3	1 (5.26%)	53 (25.11%)	
	Grade 4	2 (10.52%)	12 (5.68%)	
	Grade 5	0 (0%)	8 (3.79%)	
	Grade 6	0 (0%)	0 (0%)	
Post-operative facial nerve status (N=227)*	Grade 1	5 (26.31%)	40 (19.23%)	0.493 (Not significant)
	Grade 2	10 (52.63%)	83 (39.90%)	
	Grade 3	1 (5.26%)	48 (23.07%)	
	Grade 4	3 (15.78%)	31 (14.90%)	
	Grade 5	0 (0%)	6 (2.88%)	
	Grade 6	0 (0%)	0 (0%)	
Facial Nerve Preservation (N=227)*	Yes	18 (94.73%)	165 (79.32%)	0.104 (Not significant)
	No	1 (5.27%)	43 (20.67%)	
Facial Nerve Outcome (N=227)*	Excellent (Grade 1 or 2 )	15 (78.94%)	123 (59.13%)	0.0143 (Significant)
	Intermediate (Grade 3 or 4)	4 (21.05%)	79 (37.98%)	
	Poor (Grade 5 or 6)	0 (0%)	6 (2.88%)	
*Three patient expired on post-operative day 0 ** P-value <0.05 is considered significant				

## Discussion

There is pathological diversity in CPA tumors as they originate from various sources and abut different adjacent nerves and vascular structures. As previously discussed, CPA tumors can be categorized into two types: vestibular schwannomas (VS) and non-vestibular tumors (meningioma, epidermoid, other cranial nerve schwannoma, and cholesteatoma) [2]. Of all CPA tumors, VS accounts for 80-94%, while the remainder are non-vestibular tumors [1-3].

In this study, VS accounted for 81.7%, while the non-vestibular tumors included 8.7% meningioma, 6.5% epidermoid, and 3.1% others. Hearing loss and headache were the most common clinical features, with hearing loss observed in 100% of VS patients, 60% of meningioma patients, and 40% of epidermoid patients. Headache was reported in 90.9% of VS patients, 90% of meningioma patients, and 53.3% of epidermoid patients. Facial nerve involvement was also a prominent clinical feature, observed in 87.8% of VS patients, 40% of meningioma patients, and 26.7% of epidermoid patients. The majority of tumors in our study were either large (2.6-4 cm) or giant (> 4 cm), reflecting late presentation at the outpatient department. We achieved GTR and NTR in 93.5% of patients. CSF diversion was performed in 163 (70.86%) patients. There was a significant correlation between tumor size and facial nerve outcome, with a p-value of 0.0041. Our study found that tumor size and extent of resection were major predictive factors for facial nerve preservation (p-values of 0.0041 and 0.0442, respectively). Monitoring significantly aided facial nerve outcomes, with no poor outcomes in those monitored intra-operatively (p-value = 0.0143). CSF leaks were more common in patients with tumors larger than 4 cm. The mortality rate was 6.1% in this study, with 10 out of 14 patients who died during admission due to meningitis, respiratory failure, and hydrocephalus.

Similar histopathological distributions have been observed in other studies [2,6,9-10]. Consistent with findings by Kennedy GRS et al. [2], non-vestibular tumors were more likely to occur in the third to fourth decade of life. Unlike other studies, our study had slightly more males than females, with only meningioma showing a female predominance [2,11-16]. Kennedy GS et al., in their prospective study of 50 patients with CPA tumors, reported hearing loss as the most prominent clinical feature in 89% of cases, followed by cerebellar ataxia in 81% and headache in 74% of patients. Facial nerve and lower cranial nerve dysfunction were present in 65% and 20% of patients, respectively, in their study [2]. In comparison, Memari F et al. found that hearing loss was the most common presenting symptom in 85% of patients with CPA tumors, while 50% of patients experienced headaches and 53% had cerebellar ataxia. They reported lower percentages of facial nerve involvement (53%) and lower cranial nerve involvement (6%) in their study [15]. Similar lower incidences of facial nerve involvement in CPA tumors have been observed in other studies as well [17,18]. This difference could be attributed to the inclusion of non-vestibular tumors in these studies, whereas our study primarily involved VS patients. Similar to our study, Kennedy GS et al.'s study found that most CPA tumors (96%) were either large (2.6-4 cm) or giant (> 4 cm) [2]. Dhar S et al., in their study of 126 CPA tumors, reported an average tumor size of 3.53 cm [6].

The key point to focus on while operating on a CPA tumor is the complete removal of the tumor lesion while simultaneously preserving the functionality of the cranial nerves present in the surrounding area. It has been observed that up to 80-90% of tumor resections are possible, depending on the surgeon’s level of experience [7]. Due to the tumor’s intimate relationship with important structures of the CPA, including cranial nerves, the brainstem, and vessels, complete tumor removal is challenging without compromising these structures, especially the cranial nerves. In our study, we achieved GTR and NTR in 93.5% of patients, which was higher compared to the study by Nayak P et al. [7]. Using the retromastoid approach, Samii M and Matthies C completely removed 979 tumors; anatomical preservation of the facial nerve was achieved in 93% of patients, and of the cochlear nerve in 68% [11]. CSF diversion (VP shunt/ETV) was performed in 163 (70.86%) patients in the current study, much higher than the rates reported by Ramamurthi B [19], Kennedy GS et al. [2], and Jain VK et al. [10], which were 66%, 36%, and 8.5%, respectively.

Similar to the facial nerve preservation rate in our study, which was 80.6%, Samii M and Matthies C [11], Jain VK et al. [10], and Kennedy GS et al. [2] reported facial nerve preservation rates of 93%, 84.3%, and 67%, respectively, in their studies. Preoperatively, 44.8% of our patients had grade 2, 29.6% had grade 3 or 4, and 3.4% had grade 5 facial nerve palsy, while postoperatively, 40.9% had grade 2, 36.6% had grade 3 or 4, and 2.6% had grade 5 facial palsy, contrasting with the studies by Memari F et al. [15] and Joarder MA et al. [12]. Dhar S et al., in their study of 126 CPA tumor patients, found that the majority (71.4%) had grade 3 facial palsy preoperatively. Out of these, 42 patients experienced poor HB-grade facial nerve dysfunction immediately postoperatively, while the rest maintained similar facial nerve status [6]. In another study by Nair S et al., facial nerve function was intact in 78% of cases before surgery, but at the 6-month follow-up, 90% of cases had intermediate to poor preservation of the facial nerve. Among those with anatomically preserved facial nerves, more than half had HB grade 4 facial palsy [20].

A significant correlation was observed between the size of the tumors and the outcome of the facial nerve in this study, with poorer outcomes in patients who had tumors larger than 4 cm (88.5% vs. 71.4%, p-value = 0.0041), which was higher compared to the studies by Kennedy GS et al. (68% vs. 58%) [2] and Joarder MA et al. (74% vs. 62%) [12]. In the study by Jain VK et al., large tumors had a lower percentage of facial nerve preservation; among those with tumors larger than 4 cm, the facial nerve could be preserved in only 34.6% of patients [10]. Facial nerve preservation was higher in patients who underwent NTE and STE of tumors compared to GTE, which was statistically significant (p-value = 0.0442) in our study, consistent with previous studies [21-23]. Samii M and Matthies C reported tumor size, previous surgery or radiosurgery, and a surgeon’s operative experience as major predictive factors for facial nerve preservation [11].

CSF leaks and meningitis developed in 3.5% and 2.2% of patients in our study, respectively, far less than in the study by Kennedy GS et al. [2]. Our study's mortality rates were high compared to other studies [15,18, 24-26]. Late presentation after being treated at various clinics and the large size of tumors with hydrocephalus were major factors responsible for higher mortality in our study. The limitation of this study was that intra-operative facial nerve monitoring was not performed on all patients as indicated due to a lack of the required expertise and equipment in the initial years.

## Conclusions

The majority of CPA tumors in our study were either large or giant. Large size and late presentation with advanced disease were predictors of mortality. Preservation of facial nerve function plays a crucial role in CPA tumor surgery as it significantly affects the patient’s quality of life. Intraoperative facial nerve monitoring, a tumor size of less than 4 cm, and the extent of resection (NTR/STR) were predictive factors affecting facial nerve outcomes in this study (p-values of <0.001, 0.0041, and 0.0442, respectively).
